# Physical activity and substance use among Canadian adolescents: Examining the moderating role of school connectedness

**DOI:** 10.3389/fpubh.2022.889987

**Published:** 2022-11-10

**Authors:** Matthew James Fagan, Markus J. Duncan, Robinder P. Bedi, Eli Puterman, Scott T. Leatherdale, Guy Faulkner

**Affiliations:** ^1^University of British Columbia, Vancouver, BC, Canada; ^2^Brock University, St. Catharines, ON, Canada; ^3^University of Waterloo, Waterloo, ON, Canada

**Keywords:** physical activity, sport participation, substance use (drugs, alcohol, smoking), school connectedness, adolescence, youth

## Abstract

Physical activity may play a role in promoting or preventing substance use among youth. The purpose of this study was to examine the association between different types of physical activity [i.e., non-competitive school sport, competitive school sport, outside of school sport and minutes of moderate to vigorous physical activity (MVPA) per day] and substance use (i.e., current smoking, e-cigarette, cannabis, binge drinking) among Canadian youth. Interaction effects between physical activity and school connectedness were also examined. Using data from the COMPASS study (2018–19; *n* = 73,672), four multi-level logistic regression models were developed to investigate whether physical activity lessened or worsened the odds of (1) smoking; (2) e-cigarette use; (3) cannabis use; and (4) binge drinking. Models were stratified by gender to reflect the inherent differences between genders. Models were adjusted for demographic factors and other covariates. Sport participation was consistently associated with substance use, whereas less evidence was found for meeting MVPA guidelines. Non-competitive school sport lessened the odds of cannabis use for males and females. However, non-competitive school sport only lessened the odds of e-cigarette use for females but increased the odds of binge drinking for males. Participation in competitive school sport lessened the odds of cigarette smoking but increased the odds of e-cigarette use and binge drinking for males and females. Outside of school sport lessened the odds of cigarette smoking and cannabis use but increased the odds of e-cigarette use and binge drinking for males and females. A significant moderation effect was found for males participating in sport outside of school and meeting MVPA guidelines who were at a lower risk of e-cigarette use in the presence of high levels of school connectedness. Our study provides evidence for further consideration and provision of extracurricular activities, specifically non-competitive sport, in protecting against substance use. Caution is required in claiming that sport participation or physical activity, in general, is negatively associated with substance use among youth.

## Introduction

Many Canadians use substances that incur health risks (1–5). According to the Canadian Center for Substance Use and Addiction (CCSA), for Canadians over the age of fifteen, the top five most frequently used substances within the past year were alcohol (78.2 %), cannabis (14.8%), cocaine/crack (2.5%), hallucinogens (1.5%), and problematic prescription drug use (1.2 %) (6). The economic burden of substance use in Canada is approximately 38.4 billion dollars annually (7). The vast majority of this burden is due to alcohol (38%), tobacco (31%), opioids (9%) and cannabis (7%). The economic burden includes lost productivity, healthcare, criminal justice, and other direct costs (7). Overall, it is clear that many individuals use and potentially abuse substances, and the use of these substances directly impacts the economic burden and health of Canadians.

A particular period of research and practice interest for substance use is during adolescence. This period is typically where health behaviors, particularly substance use, are adopted and cemented (8–10). Substance use during adolescence is highly associated with adverse health and social outcomes during adolescence and into adulthood. For example, substance use during adolescence has been associated with lower grades, delinquency and cognitive impairment (11). When considering the long-term effects of adolescent substance use, it has been longitudinally associated with trouble with the law, substance use disorders, and adverse health outcomes into adulthood (12). The importance of examining the complexity of the etiology of substance use during adolescence should not be understated.

Many theories and models have attempted to explain experimental substance use in adolescence (13). Petraitis et al. (13) summarized these existing models and theories into one succinct framework. They indicate three major categories of factors and three major levels of factors (creating a 3-category x 3-level conceptual framework) for the etiology of experimental substance use. In terms of the categories, Petraitis et al. (13) identified (1) interpersonal/social (e.g., interplay between people), (2) cultural/attitudinal (e.g., how immediate surroundings shape attitudes), and (3) intrapersonal factors (e.g., unique to an individual). The three levels of influence consist of ultimate (e.g., out of the control of the individual), distal (indirect factors) and proximal factors (e.g., direct factors) (13). For a full breakdown of the constructs included in this conceptual framework, please refer to the article by Petraitis et al. (13). Utilizing this conceptual framework may be an appropriate starting point in testing various constructs of substance use in adolescents.

Inadequate physical activity is another modifiable risk factor common among adolescents (14). It is important to separate the umbrella term of physical activity into different categories. Physical activity is defined as any bodily movement that incurs energy expenditure (15). A subset of physical activity is exercise, defined as energy expenditure for gaining or maintaining fitness (15). Finally, another subset of physical activity is sport participation which is rule-governed and structured (16). These distinct but overlapping definitions are important to consider as evidence suggests differential associations with substance use based on how physical activity is conceptualized and measured. Within the 24-h movement guidelines for Canadian children and adolescents, it is recommended that children and adolescents accumulate at least 60 min of moderate to vigorous physical activity (MVPA) per day (17). Overall, exercise and minutes of MVPA have been shown to be negatively associated with the use of several substances among adolescents, including cigarette smoking, cannabis and illicit drug use (18, 19). However, regarding alcohol consumption, the evidence for a negative association with physical activity is less clear, as physical activity encompasses incidental movement, exercise and sport, making it difficult to untangle how physical activity participation might be protective or risky in terms of substance use (19). Furthermore, alcohol consumption, and more specifically, binge drinking, is more common among adolescents active in extracurricular activities like sports (20).

A recent publication by Williams et al. (21) examined the association between sport participation and substance use in Canadian adolescents. They found unique associations with substance use depending on the type of sport participation that was being examined (non-competitive school and competitive school sport). Non-competitive school sport participation was negatively associated with cigarette smoking and cannabis for females and males and e-cigarette use for females. However, competitive school sport participation was positively associated with alcohol use and e-cigarette use for males and females (21). A review by Lisha and Sussman (22) indicates that sport participation was predominately negatively associated with cigarette use and illicit drug use while being a risk factor for alcohol use. The differences in results for sport participation may be due to the different types of sport (individual vs. team) and levels (e.g., non-competitive school vs. competitive; school vs. community-based) that could be played, and the psychosocial mechanisms may be different depending on these variations in sport types (21, 23). Finally, much like the physical activity literature, prospective studies have provided evidence for the preceding nature of sport participation and its association with substance use (24, 25).

Additionally, as described in the conceptual framework by Petraitis et al. (13), important factors may moderate the association between physical activity and experimental substance use. One, in particular, that may be playing a role is school connectedness. As others have noted, a consistent definition of school connectedness is lacking and at times overlaps with other important concepts (e.g., school climate) (26). School connectedness can be defined as a student's perception of how adults in their school care for their success in school and as an individual (27). However, based on the inconsistency within the literature, the global concept of school connectedness is typically considered feelings toward school, teachers and classmates (26). This has been identified as a potential moderator in the Petraitis et al.'s model (13) (cultural/attitudinal category and distal level) and is associated with adolescent substance use in the literature (28). Drawing from the Social Developmental Model, school connectedness theoretically enhances a social bond between the student and school, which in turn “inhibits behaviors inconsistent with the beliefs held and behaviors practiced by the socialization unit [the school] through establishment of an individual's stake in conforming to its norms, values and behaviors” [(29), p 252]. Individuals with high school connectedness may be at less risk of substance use when the behavior is discouraged or is not perceived as normative within the group setting. For example, it is possible that school sport participation exposes the adolescents to role models (including peers and teachers) from the school and fosters a sense of belongingness to the institution. Additionally, students that possess high levels of school connectedness may be less at risk for substance use than their sport playing peers as they may have different normative values. However, to the authors' knowledge, whether school connectedness moderates the association between physical activity participation and substance use has not been assessed.

Therefore, the objectives of this cross-sectional study are to examine the association between MVPA and sport participation and substance use among Canadian adolescents, and explore if school connectedness moderates these associations. More specifically, we examine associations between meeting the MVPA guideline outlined by the Canadian 24-h movement guidelines (17) and different types of sport participation (i.e., competitive school sport, non-competitive school sport participation, and outside-of-school sport participation) when models are created for specific types of substance use (i.e., current cigarette smoking, current e-cigarette smoking, current cannabis use and current binge drinking). Within these models, we also examine if school connectedness moderates the associations with an expectation that school connectedness may attenuate engagement in substance use.

## Methods

The analysis uses data from the COMPASS study, an ongoing cohort study (2012–2021) that collects hierarchical and linked longitudinal behavioral and program/policy data in Canadian adolescents. The 2018–19 school year was used, including 73,672 students in grades 9–12 (Secondary I–IV in Quebec). The schools consist of *n* = 30,301 from Ontario, *n* = 29,904 from Quebec, *n* = 10,238 from British Columbia, and *n* = 3,261 from Alberta. The COMPASS system was created for evaluating natural experiments and therefore was not designed to be representative of all Canadian adolescents (30). For a complete description of the COMPASS study, please see Leatherdale and colleagues (30) or online at www.compass.uwaterloo.ca. The study was approved by the University of Waterloo Office of Research Ethics, UBC's research ethics board, and appropriate school board committees.

### Data collection tools

The student-level questionnaire collected all data except the median school income. Median school income was determined by the sortation area (the first three alphanumeric digits of the postal code for the school). The student-level questionnaire collects data about the students' demographics, behaviors and correlates the behaviors. All measures used in the COMPASS study are consistent with other surveillance systems (31, 32).

### Measures

#### Substance use behaviors

Consistent with previous work (21), our analysis created four dichotomous substance use measures to reflect current use (i.e., using a substance at least once a month). Therefore, a dichotomous variable of current cigarette smoking was created from answering at least ‘one day’ to the question “on how many of the last 30-days did you smoke or more cigarettes?” with ‘1 day or more' being coded as current, and ‘none or never’ being coded as non-use. For e-cigarette use, a dichotomous variable of current use was created from answering at least ‘1 day' to the question “On how many of the last 30 days did you use an e-cigarette?” with ‘1 day or more' being coded as current use and ‘none or never’ being coded as non-use. A dichotomous current binge drinking variable was created by answering at least ‘once a month’ to the question “in the last 12 months, how often did you have five drinks of alcohol or more on one occasion?” with ‘1 day a month or more' being coded as current use and ‘none or never’ being coded as non-use. Finally, current cannabis use was created by answering ‘at least once a month’ to the question “in the last 12 months, how often did you use marijuana or cannabis?” with ‘once a month’ or more being coded as current use and ‘none or never’ being coded as non-use.

#### Physical activity and sport participation

Consistent with other publications (21, 33, 34), separate dichotomous sport participation measures are used in the analysis—first, participation in non-competitive school sports (e.g., intramural sport). Second, participation in competitive school sports (e.g., varsity sports) and, finally, outside-of-school sports (e.g., sports league outside-of-school). One measure for physical activity was used in the analysis. A dichotomous variable of meeting the 60 min of recommended MVPA each day was created in line with Canada's 24-h movement guidelines (35). The physical activity measure within the COMPASS questionnaire has acceptable test-retest reliability and criterion validity (36).

#### School connectedness

School connectedness was collected by utilizing a modified version of the National Longitudinal Study of Adolescent Health School Connectedness scale (37). The items within this scale attempt to tap into global school connectedness by capturing the belonging, liking/enjoyment, closeness, fair treatment, and safety felt by the child. These items directly capture relationships with teachers through the question (“I feel the teachers at my school treat me fairly”). The remaining questions probe belongingness (“I feel I am a part of my school”), school enjoyment (“I am happy to be at my school”), closeness (“I feel close to people at my school”), safety (“I feel safe in my school”) and the importance of getting good grades (“Getting good grades is important to me”). These questions are thought to implicitly address relationships with teachers and peers (26, 28). These items are summed into a unidimensional measure that ranges from 6–24 with higher scores indicating higher levels of school connectedness. These items have been used in the Youth Smoking Survey to assess school connectedness (38).

#### Covariates

Several covariates were included in the models, based on previous research identifying associations between them and either substance use behaviors, physical activity/sport participation or both: grade (9–12), pocket money (none, 1–20$, 21–100$, >100$, I don't know), ethnicity (white, black, Asian, Hispanic/Latin, other and mixed ethnicity), anxiety (none, mild, moderate and severe), and school connectedness. Anxiety was collected by the Generalized Anxiety Disorder Assessment-7 (GAD-7) and following the recommendations from the GAD-7 scoring 0–4 as none, 5–9 as mild, 10–14 as moderate, and 15–21 as severe (39). Gender (male, female) was only used as a covariate in the unstratified sample. Additionally, all other substance use, sport participation, and physical activity variables were included as covariates in all models.

### Statistical analysis

Descriptive statistics were used to determine demographic and substance use differences (Student's *t*-test for continuous variables and chi-square test for categorical variables). All analyses were run using *Rstudio* (40) with the *lme4* package (41) to build the multi-level logistic regressions models with a random intercept accounting for the differing school identification codes. As key variables (e.g., substance use and physical activity) included in the analysis differed by gender, the analysis was stratified. As indicated by the Canadian Institute of Health Research (CIHR) Institute of Gender and Health, accounting for gender within secondary data analysis is important (42). One of their primary suggestions include stratifying the dataset (43). This would assume differences among men and women in health behaviors given societal impact (e.g., patriarchal society normalizes sport participation for men more so than women) and key variables differ by gender (substance use patterns and physical activity).

Using the package *lme4*, four separate multi-level models were created for (1) current cigarette use, (2) current e-cigarette use, (3) current binge drinking, and (4) current cannabis use. The final non-interaction multi-level logistical regressions were built in 5 stages; (1) the base model included only the random intercept effects and outcome of interest (intraclass correlation coefficient (ICC) used to determine the amount of variance accounted for by the random intercept from the school level). (2), only the predictor of interest was inputted into the model, followed by (3) demographic covariates (grade, gender (only unstratified sample), ethnicity, weekly spending money (proxy for SES)), (4) social and mental health variables (anxiety score, other substance use) and (5) controlling for other sport participation and MVPA. The AIC was used to determine progression in model fit. The final non-moderation models utilized all variables outlined above. For the permutation interaction models, the moderator of interest (e.g., school connectedness and its interaction with sport participation) was inputted into each model. The *p*-values were adjusted for false discovery rate (FDR) with the Benjamini-Hochberg (BH) method by the *p. adjust(p, method* = “*BH”)* function in the *stats* package (44–47). It was applied to the final model, including moderation analysis. After adjustments, the significance was accepted at *p* ≤ 0.05.

## Results

### Demographics and descriptive results

The sample included (~50.2%) males and the majority identified as white (~69%). Full demographic results by gender are presented in Table 1. Significant gender differences were identified for current smoking [7% of females (f) vs. 8% of males (m)], current binge drinking (16% f vs. 19% m), current e-cigarette use (26% f vs. 31% m), current cannabis use (11% f vs. 15% m), participation in non-competitive school sport (34% f vs. 38% m), participation in competitive school sport (33% f vs. 40% m), participation in outside of sport (37% f vs. 45% m), and self-reported ≥ 60 min of MVPA (31% f vs. 46% m).

**Table 1:** Demographic and descriptive statistics.

**Variable**	**Total**	**Females, *N* = 36,546**	**Males, *N* = 37,126**	***P*-value**
**Grade**				0.009
9	17,182 (29%)	8,402 (28%)	8,780 (29%)	
10	17,112 (29%)	8,659 (29%)	8,453 (28%)	
11	15,849 (27%)	7,938 (27%)	7,911 (26%)	
12	9,292 (14%)	4,574 (15%)	4,718 (16%)	
**Ethnicity**				<0.001
White	50,860 (70%)	25,383 (70%)	25,477 (69%)	
Mixed	5745 (7.8%)	3,032 (8.3%)	2,713 (7.3%)	
Black	2909 (4%)	1,264 (3.5%)	1,645 (4.4%)	
Asian	7431 (10%)	3,726 (10%)	3,705 (10%)	
Latin	1874 (2.5%)	902 (2.5%)	972 (2.6%)	
Other	4,609 (6.3%)	2,139 (5.9%)	2,470 (6.7%)	
**Province**				0.002
Alberta	3,261 (4.5%)	1,629 (4.5%)	1,632 (4.4%)	
British Columbia	10,238 (14%)	4,989 (14%)	5,249 (14%)	
Ontario	30,269 (41.5%)	14,852 (41%)	15,417 (42%)	
Quebec	29,904 (40%)	15,076 (41%)	14,828 (40%)	
**Pocket money (SES proxy)**				<0.001
Zero	11,586 (16%)	5,123 (14%)	6,463 (18%)	
1–20$	17,684 (24.5%)	9,030 (25%)	8,654 (24%)	
21–100$	16,718 (22.5%)	8,881 (24%)	7,837 (21%)	
>100$	14,107 (19%)	6,395 (18%)	7,712 (21%)	
I don't know	12,920 (18%)	6,830 (19%)	6,090 (17%)	
**Participation in non-competitive school sport**				<0.001
No	44,753 (64%)	23,042 (66%)	21,711 (62%)	
Yes	25,428 (36%)	11,940 (34%)	13,488 (38%)	
**Participation in competitive school sport**				<0.001
No	45,581 (63.5%)	24,107 (67%)	21,474 (60%)	
Yes	26,038 (36.5%)	11,616 (33%)	14,422 (40%)	
**Participation in outside of school sport**				<0.001
No	42,064 (61.5%)	22,387 (63%)	19,677 (55%)	
Yes	29,775 (39.5%)	13,393 (37%)	16,382 (45%)	
**Greater than or equal to 60 mins of MVPA per day**				<0.001
No	44,179 (61.5%)	24,712 (69%)	19,467 (54%)	
Yes	27,861 (39.5%)	11,151 (31%)	16,710 (46%)	
**Current e-cigarette use**				<0.001
No	52,188 (71.5%)	26,951 (74%)	25,237 (69%)	
Yes	20,576 (29.5%)	9,312 (26%)	11,264 (31%)	
**Current cigarette smoking**				<0.001
No	67,739 (92.5%)	33,949 (93%)	33,790 (92%)	
Yes	5,394 (7.5%)	2,425 (6.7%)	2,969 (8.1%)	
**Current cannabis use**				<0.001
No	63,133 (86.5%)	32,183 (89%)	30,950 (85%)	
Yes	9,459 (13.5%)	4,001 (11%)	5,458 (15%)	
**Current binge drink behaviour**				<0.001
No	60,753 (82.5%)	30,733 (84%)	30,020 (81%)	
Yes	12,678 (17.5%)	5,703 (16%)	6,975 (19%)	
**Ever use opiates**				<0.001
No	70,273 (99%)	35,363 (99%)	34,910 (99%)	
Yes	687 (1%)	208 (0.6%)	479 (1.4%)	
**School connectedness**	18.0 (17.0,21.0)	18.0 (17.0, 20.0)	19.0 (17.0, 21.0)	<0.001
**General Anxiety Disorder-7**				<0.001
0–4 (none)	32,974 (49%)	12,084 (36%)	20,890 (62%)	
5–9 (mild)	18,617 (27.5%)	10,542 (31%)	8,075 (24%)	
9–14 (moderate)	8,991 (13.5%)	5,952 (18%)	3,039 (9.0%)	
15–21 (severe)	7,093 (10%)	5,167 (15%)	1,926 (5.7%)	

n (%); Median (IQR), p-values; Pearson's Chi-squared test; Wilcoxon rank sum test, SES, social economic status; MVPA, moderate to vigorous physical activity.

### Regression analysis

For breakdown AIC during model building and ICC to reflect random effects (school clustering) for current cigarette, e-cigarette and cannabis use and binge drinking please see Tables S1–S4 in the supplementary file.

#### Model 1 cigarette use

For females, participation in competitive school sports and sport outside of school were associated with 28 and 27% lower odds of current cigarette use. A similar reduction in odds was found for males participating in competitive school sport (33%) and sport outside of school (28%). No associations were identified for non-competitive school sport or meeting the MVPA guidelines for females or males. For a full model summary see Table 2.

**Table 2:** Full model summaries for current cigarette smoking.

**Characteristic**	**OR**	**95% CI**	**OR**	**95% CI**	**OR**	**95% CI**
**Stratified**	**Female**		**Male**		**Full sample**	
**Grade**						
9	–	–	–	–	–	–
10	1.03	0.86, 1.24	1.02	0.85, 1.22	1.03	0.90, 1.17
11	1.15	0.96, 1.39	**1.22**	**1.03, 1.46**	**1.20**	**1.06, 1.36**
12	**1.34**	**1.08, 1.65**	**1.53**	**1.26, 1.87**	**1.45**	**1.26, 1.68**
**Gender**						
**Female**					–	–
**Male**					1.06	0.97, 1.16
**Ethnicity**						
White	–	–	–	–	–	–
Mixed	1.24	1.00, 1.54	**1.31**	**1.05, 1.63**	**1.29**	**1.10, 1.50**
Black	0.78	0.50, 1.21	**1.60**	**1.19, 2.16**	1.27	1.00, 1.63
Asian	**0.62**	**0.45, 0.85**	1.15	0.88, 1.51	0.88	0.71, 1.08
Latin	1.13	0.76, 1.68	1.27	0.89, 1.81	1.23	0.95, 1.60
Other	**2.04**	**1.64, 2.54**	**1.97**	**1.59, 2.43**	**1.95**	**1.67, 2.27**
**Pocket money**						
None	–	–	–	–	–	–
1–20$	1.00	0.78, 1.27	1.08	0.85, 1.37	1.05	0.89, 1.25
21–100$	0.91	0.72, 1.15	1.10	0.88, 1.37	1.01	0.86, 1.18
>100$	0.94	0.74, 1.20	**1.28**	**1.03, 1.59**	1.11	0.95, 1.30
“I don't know”	0.90	0.69, 1.17	1.08	0.84, 1.40	1.00	0.83, 1.20
**GAD-7**						
0 (minimal)	–	–	–	–	–	–
1 (mild)	1.12	0.94, 1.34	**1.19**	**1.04, 1.37**	**1.16**	**1.04, 1.29**
2 (Moderate)	1.09	0.90, 1.33	**1.36**	**1.13, 1.65**	**1.21**	**1.06, 1.38**
3 (severe)	**1.38**	**1.14, 1.67**	**1.64**	**1.32, 2.02**	**1.52**	**1.33, 1.75**
**School connectedness**	**0.89**	**0.87, 0.91**	**0.95**	**0.93, 0.96**	**0.92**	**0.91, 0.94**
**Current e-cigarette use**						
No	–	–	–	–	–	–
Yes	**5.25**	**4.52, 6.10**	**7.59**	**6.42, 8.98**	**6.19**	**5.54, 6.92**
**Current cannabis use**						
No	–	–	–	–	–	–
Yes	**4.77**	**4.16, 5.47**	**3.93**	**3.45, 4.48**	**4.36**	**3.97, 4.79**
**Current binge drinking**						
No	–	–	–	–	–	–
Yes	**2.52**	**2.19, 2.89**	**2.63**	**2.31, 3.00**	**2.56**	**2.33, 2.81**
**Ever-use opiates**						
No	–	–	–	–	–	–
Yes	**6.19**	**3.64, 10.5**	**4.75**	**3.41, 6.62**	**5.05**	**3.82, 6.67**
**Outside of school sport**						
No	–	–			–	–
Yes	**0.73**	**0.63, 0.85**	**0.72**	**0.63, 0.82**	**0.73**	**0.66, 0.81**
**Competitive school sport**						
No	–	–	–	–	–	–
Yes	**0.72**	**0.61, 0.86**	**0.67**	**0.57, 0.78**	**0.70**	**0.63, 0.79**
**MVPA guidelines**						
No	–	–	–	–	–	–
Yes	1.12	0.98, 1.28	1.13	1.00, 1.27	**1.13**	**1.03, 1.23**
**Non-competitive school sport**						
No	–	–	–	–	–	–
Yes	0.86	0.72, 1.02	0.88	0.76, 1.03	**0.87**	**0.78, 0.98**

OR, odds ratio; CI, confidence interval, Bold, BH adjusted p-value < 0.05. MVPA, moderate to vigorous physical activity. GAD-7, generalized anxiety disorder-7.

**Figure 1:**
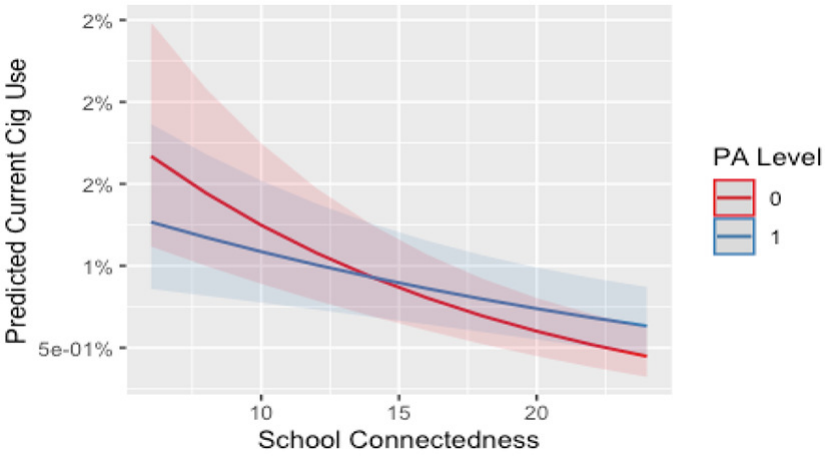
Interaction between school connectedness and MVPA level on current cigarette use for males. 0, no; 1, yes. PA level, meeting moderate to vigorous physical activity level based on Canadian guidelines. Line and shade, mean and SE.

There was no significant interaction between school connectedness and sport participation or self-reported MVPA for females. For males who were highly connected to school, meeting the recommended minutes of MVPA was associated with a 4% increased odds of current cigarette smoking. However, based on Figure 1, both participants and non-participants saw a protective effect from school connectedness. For full model summary of interaction model see Table S5 in the supplementary file.

#### Model 2 e-cigarette use

For females, participation in non-competitive school sport was associated with a 11% lower odds of e-cigarette use. However, competitive school sport and outside of school sport were associated with a 42 and 10% higher odds of e-cigarette use. Meeting the MVPA guidelines was not associated with e-cigarette use for females. For males, participation in competitive school sport, outside of school sport and meeting the MVPA guidelines were associated with a 41, 29, and 15% higher odds of current e-cigarette use, respectively. Non-competitive school sport was not associated with e-cigarette use. See Table 3 for full model summary.

**Table 3:** Full model summaries for current e-cigarette use.

**Characteristic**	**OR**	**95% CI**	**OR**	**95% CI**	**OR**	**95% CI**
**Stratified**	**Female**		**Male**		**Full sample**	
**Grade**						
9	–	–	–	–	–	–
10	**1.10**	**1.01, 1.20**	1.07	0.98, 1.17	**1.09**	**1.02, 1.16**
11	0.89	0.81, 0.98	1.06	0.96, 1.16	0.98	0.91, 1.04
12	0.86	0.76, 0.97	**0.87**	**0.78, 0.98**	**0.89**	**0.81, 0.96**
**Gender**						
Female					–	–
Male					**1.33**	**1.26, 1.39**
**Ethnicity**						
White	–	–	–	–	–	–
Mixed	0.94	0.83, 1.06	**0.82**	**0.71, 0.94**	**0.90**	**0.82, 0.98**
Black	**0.58**	**0.46, 0.72**	**0.60**	**0.49, 0.72**	**0.60**	**0.52, 0.69**
Asian	**0.51**	**0.44, 0.59**	**0.58**	**0.50, 0.66**	**0.55**	**0.50, 0.62**
Latin	0.93	0.75, 1.14	0.93	0.76, 1.14	0.94	0.81, 1.09
Other	**0.73**	**0.63, 0.86**	**0.79**	**0.68, 0.91**	**0.77**	**0.70, 0.86**
**Pocket money**						
None	–	–	–	–	–	–
1–20$	**1.76**	**1.54, 2.00**	**1.45**	**1.29, 1.64**	**1.58**	**1.45, 1.72**
21–100$	**2.41**	**2.12, 2.73**	**1.93**	**1.72, 2.17**	**2.14**	**1.96, 2.33**
>100$	**2.75**	**2.41, 3.13**	**2.20**	**1.96, 2.48**	**2.42**	**2.22, 2.64**
“I don't know”	**1.45**	**1.26, 1.67**	**1.33**	**1.17, 1.52**	**1.38**	**1.25, 1.51**
**GAD-7**						
0 (minimal)	–	–	–	–	–	–
1 (mild)	**1.38**	**1.26, 1.50**	**1.36**	**1.25, 1.47**	**1.34**	**1.27, 1.42**
2 (Moderate)	**1.64**	**1.49, 1.81**	**1.24**	**1.10, 1.40**	**1.46**	**1.36, 1.58**
3 (severe)	**1.53**	**1.37, 1.70**	**1.21**	**1.04, 1.41**	**1.40**	**1.29, 1.53**
**School connectedness**	**0.97**	**0.96, 0.98**	**0.98**	**0.97, 0.99**	**0.98**	**0.97, 0.98**
**Current cigarette use**						
No	–	–	–	–	–	–
Yes	**4.45**	**3.82, 5.18**	**6.47**	**5.45, 7.69**	**5.22**	**4.66, 5.84**
**Current cannabis use**						
No	–	–	–	–	–	–
Yes	**4.41**	**3.97, 4.91**	**7.21**	**6.49, 8.00**	**5.68**	**5.27, 6.11**
**Current binge drinking**						
No	–	–	–	–	–	–
Yes	**4.78**	**4.39, 5.20**	**4.50**	**4.13, 4.91**	**4.61**	**4.34, 4.89**
**Ever-use opiates**						
No	–	–	–	–	–	–
Yes	0.77	0.43, 1.37	1.02	0.65, 1.60	0.92	0.65, 1.31
**Outside of school sport**						
No	–	–	–	–	–	–
Yes	**1.10**	**1.02, 1.18**	**1.29**	**1.19, 1.39**	**1.19**	**1.13, 1.26**
**Competitive school sport**						
No	–	–	–	–	–	–
Yes	**1.42**	**1.30, 1.55**	**1.41**	**1.29, 1.54**	**1.42**	**1.34, 1.51**
**MVPA guidelines**						
No	–	–	–	–	–	–
Yes	1.02	0.95, 1.10	**1.15**	**1.08, 1.24**	**1.10**	**1.05, 1.16**
**Non-competitive school sport**						
No	–	–	–	–	–	–
Yes	**0.89**	**0.82, 0.97**	0.92	0.85, 1.00	**0.90**	**0.85, 0.96**

OR, odds ratio, CI, confidence interval, Bold, BH adjusted p-value < 0.05. MVPA, moderate to vigorous physical activity. GAD-7, generalized anxiety disorder-7.

**Figure 2:**
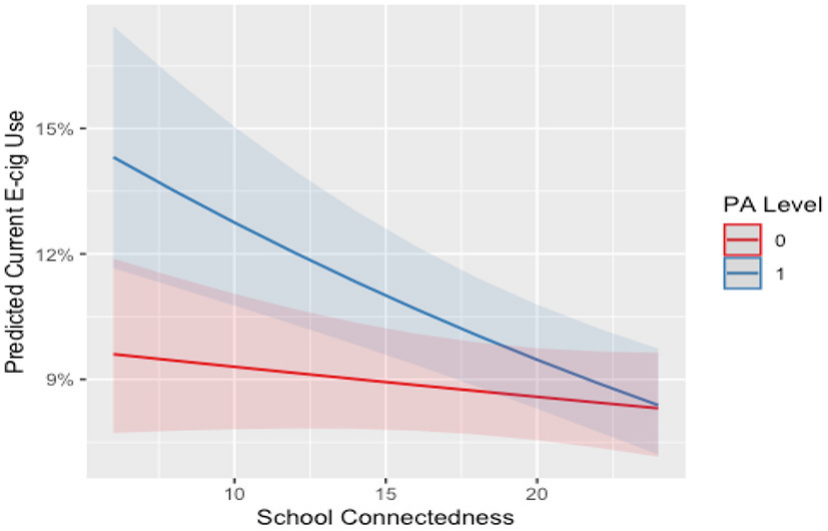
Interaction between school connectedness and outside of school sport on current E-cigarette use for males. E-cig, E-cigarette; 0, not participating; 1, participating. Line and shade, mean and SE.

**Figure 3:**
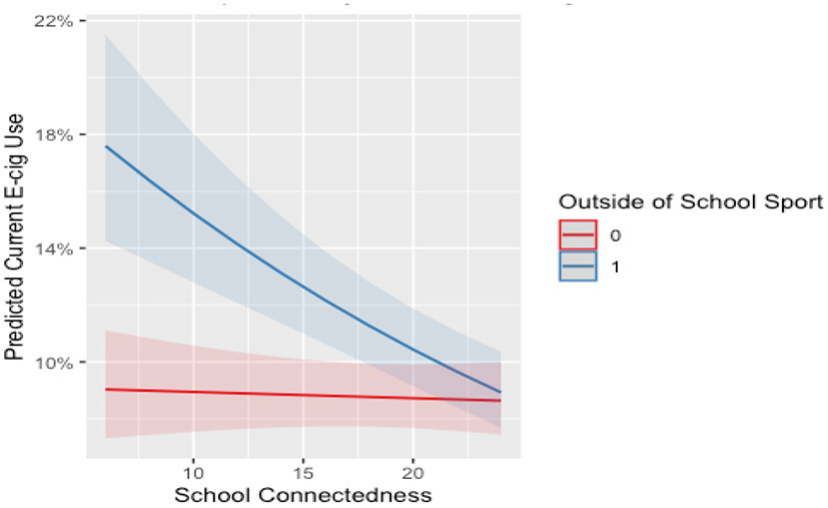
Interaction between school connectedness and MVPA level E-cigarette use for males. E-cig, E-cigarette; 0, not participating; 1, participating; PA level, meeting the MVPA guidelines. Line and shade, mean and SE.

There was no significant interaction between school connectedness and sport participation or self-reported MVPA for females. For males who were highly connected to school, participation in outside of school sport or meeting the MVPA guidelines were associated with a 4 and 2% lower odds of current e-cigarette use. For a visualization of the interaction effects please see Figures 2, 3. For a full summary of the interaction model see Tables S6, S7 in the supplementary file.

#### Model 3 cannabis use

For females, participation in non-competitive school sport and sport outside of school were associated with 20 and 22% lower odds of current cannabis use. Participation in competitive school sport and meeting the MVPA guidelines were not associated with current cannabis use. For males, non-competitive school sport was associated with a 17% lower odds of current cannabis use. Participation in competitive school, outside of school sport and meeting the MVPA guidelines were not associated with current cannabis use. See Table 4 for full model summary.

**Table 4:** Full model summaries for current cannabis use.

**Characteristic**	**OR**	**95% CI**	**OR**	**95% CI**	**OR**	**95% CI**
**Stratified**	**Female**		**Male**		**Full sample**	
**Grade**						
9	–	–	–	–	–	–
10	**1.29**	**1.11, 1.49**	**1.40**	**1.23, 1.60**	**1.33**	**1.21, 1.47**
11	**1.66**	**1.43, 1.91**	**1.68**	**1.47, 1.92**	**1.66**	**1.51, 1.83**
12	**1.90**	**1.61, 2.24**	**2.25**	**1.94, 2.62**	**2.01**	**1.80, 2.25**
**Gender**						
Female						–
Male					**1.46**	**1.36, 1.57**
**Ethnicity**						
White	–	–	–	–	–	–
Mixed	1.15	0.97, 1.36	**1.29**	**1.09, 1.52**	**1.19**	**1.06, 1.34**
Black	**1.58**	**1.22, 2.06**	**1.83**	**1.47, 2.28**	**1.68**	**1.42, 1.99**
Asian	**0.50**	**0.40, 0.63**	**0.57**	**0.46, 0.70**	**0.53**	**0.45, 0.62**
Latin	0.88	0.65, 1.18	0.97	0.74, 1.27	0.91	0.74, 1.11
Other	**1.51**	**1.25, 1.83**	**1.65**	**1.39, 1.96**	**1.56**	**1.37, 1.77**
**Pocket money**						
None	–	–	–	–	–	–
1–20$	**1.43**	**1.18, 1.74**	1.18	0.99, 1.40	**1.30**	**1.15, 1.48**
21–100$	**1.48**	**1.23, 1.79**	**1.50**	**1.28, 1.77**	**1.49**	**1.32, 1.68**
>100$	**1.59**	**1.32, 1.92**	**1.53**	**1.30, 1.79**	**1.56**	**1.38, 1.77**
“I don't know”	1.09	0.88, 1.36	1.08	0.89, 1.31	1.11	0.96, 1.27
**GAD-7**						
0 (minimal)	–	–	–	–	–	–
1 (mild)	**1.22**	**1.07, 1.40**	**1.14**	**1.02, 1.26**	**1.14**	**1.05, 1.24**
2 (Moderate)	**1.44**	**1.24, 1.66**	**1.26**	**1.09, 1.47**	**1.31**	**1.19, 1.45**
3 (severe)	**1.63**	**1.40, 1.89**	**1.33**	**1.11, 1.58**	**1.49**	**1.34, 1.66**
**School connectedness**	**0.90**	**0.88, 0.91**	**0.93**	**0.91, 0.94**	**0.92**	**0.91, 0.93**
**Current cigarette use**						
No	–	–	–	–	–	–
Yes	**4.72**	**4.12, 5.42**	**3.77**	**3.30, 4.30**	**4.24**	**3.86, 4.66**
**Current e-cigarette use**						
No	–	–	–	–	–	–
Yes	**4.96**	**4.46, 5.51**	**7.79**	**7.02, 8.65**	**6.21**	**5.77, 6.69**
**Current binge drinking**						
No	–	–	–	–	–	–
Yes	**4.16**	**3.74, 4.63**	**3.32**	**3.00, 3.67**	**3.75**	**3.48, 4.03**
**Ever-use opiates**						
No	–	–	–	–	–	–
Yes	**11.90**	**5.80, 24.60**	**6.87**	**4.38, 10.80**	**7.97**	**5.43, 11.70**
**Outside of school sport**						
No	–	–	–	–	–	–
Yes	**0.78**	**0.70, 0.87**	0.90	0.82, 1.00	**0.85**	**0.79, 0.91**
**Competitive school sport**						
No	–	–	–	–	–	–
Yes	1.01	0.89, 1.16	1.04	0.93, 1.17	1.03	0.94, 1.12
**MVPA guidelines**						
No	–	–	–	–	–	–
Yes	1.09	0.98, 1.21	1.09	0.99, 1.20	**1.08**	**1.01, 1.16**
**Non-competitive school sport**						
No	–	–	–	–	–	–
Yes	**0.80**	**0.70, 0.91**	**0.83**	**0.74, 0.93**	**0.82**	**0.75, 0.89**

OR, odds ratio, CI, confidence interval, Bold, BH adjusted p-value < 0.05. MVPA, moderate to vigorous physical activity. GAD-7, generalized anxiety disorder-7.

**Figure 4:**
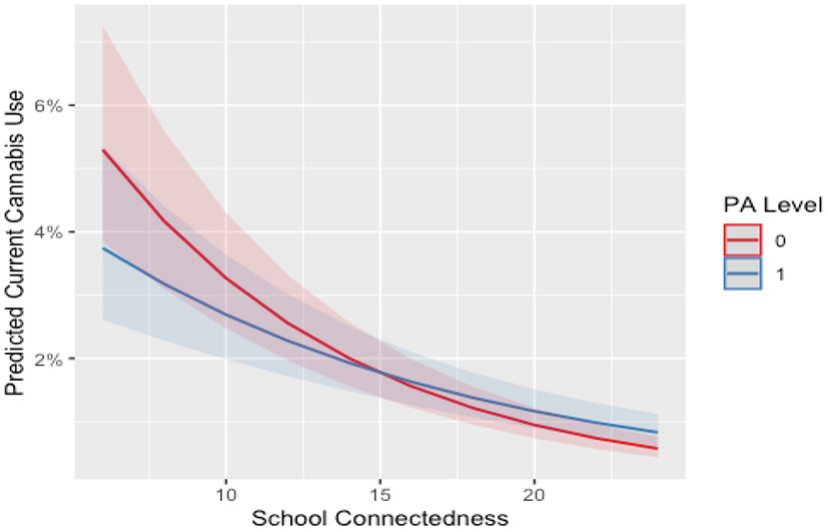
Interaction between school connectedness and MVPA level on current cannabis use for females. 0, no, 1, yes.PA level, meeting moderate to vigorous physical activity level based on Canadian guidelines. Line and shade, mean and SE.

**Figure 5:**
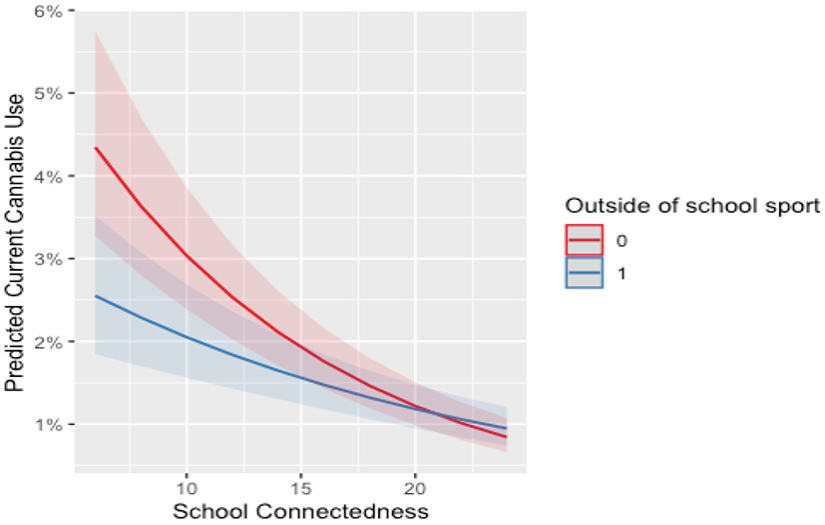
Interaction between school connectedness and outside of school sport on current cannabis use for males. 0, not participating; 1, participating. Line and shade, mean and SE.

There was no significant interaction between school connectedness and sport participation for females. For females who were highly connected to school, meeting the MVPA guidelines increased the odds of current cannabis use by 4%. For males who were highly connected to school, participation in outside of school sport was associated with 4% higher odds of current cannabis use. However, based on Figures 4, 5 both participants and non-participants saw a protective effect from school connectedness. For full model summaries see Tables S8, S9 in the supplementary file.

#### Model 4 binge drinking

For females, participation in competitive school sport, sport outside of school and meeting the recommend MVPA guideline were associated with 47, 18, and 10% higher odds of current binge drinking respectively. Non-competitive school sport was not associated with binge drinking. For males, participation in non-competitive school sport, competitive school sport, outside of school sport, and meeting the MVPA guidelines were associated with 18, 49, 38, and 24% higher odds of current binge drinking respectively. For full model summaries see Table 5.

**Table 5:** Full model summaries for current binge drinking.

**Characteristic**	**OR**	**95% CI**	**OR**	**95% CI**	**OR**	**95% CI**
**Stratified**	**Female**		**Male**		**Full sample**	
**Grade**						
9	–	–	–	–	–	–
10	**1.55**	**1.38, 1.74**	**1.66**	**1.48, 1.86**	**1.60**	**1.48, 1.74**
11	**2.12**	**1.88, 2.39**	**2.42**	**2.16, 2.71**	**2.27**	**2.09, 2.46**
12	**2.77**	**2.40, 3.20**	**3.32**	**2.89, 3.82**	**3.10**	**2.80, 3.43**
**Gender**						
Female					–	–
Male					1.02	0.96, 1.08
**Ethnicity**						
White	–	–	–	–	–	–
Mixed	**0.75**	**0.64, 0.88**	0.95	0.81, 1.11	**0.86**	**0.77, 0.96**
Black	**0.59**	**0.44, 0.79**	**0.63**	**0.50, 0.79**	**0.63**	**0.53, 0.76**
Asian	**0.51**	**0.42, 0.63**	**0.71**	**0.59, 0.86**	**0.63**	**0.55, 0.72**
Latin	**1.26**	**0.98, 1.61**	1.17	0.91, 1.49	**1.24**	**1.04, 1.48**
Other	**0.80**	**0.66, 0.96**	**0.66**	**0.56, 0.80**	**0.73**	**0.64, 0.83**
**Pocket money**						
None	–	–	–	–	–	–
1–20$	**1.30**	**1.09, 1.54**	**1.24**	**1.06, 1.45**	**1.28**	**1.14, 1.43**
21–100$	**1.88**	**1.60, 2.21**	**1.79**	**1.54, 2.08**	**1.81**	**1.62, 2.02**
>100$	**2.15**	**1.82, 2.53**	**2.28**	**1.97, 2.64**	**2.18**	**1.95, 2.43**
“I don't know”	**1.27**	**1.06, 1.53**	**1.37**	**1.15, 1.61**	**1.31**	**1.16, 1.49**
**GAD-7**						
0 (minimal)	–	–	–	–	–	–
1 (mild)	**1.17**	**1.05, 1.30**	0.98	0.89, 1.07	1.05	0.98, 1.12
2 (Moderate)	**1.14**	**1.02, 1.29**	0.88	0.77, 1.02	1.02	0.93, 1.12
3 (severe)	1.11	0.98, 1.27	1.03	0.87, 1.23	1.07	0.97, 1.18
**School connectedness**	**0.98**	**0.96, 0.99**	0.99	0.98, 1.01	**0.99**	**0.98, 0.99**
**Current cigarette use**						
No	–	–	–	–	–	–
Yes	**2.25**	**1.96, 2.59**	**2.42**	**2.12, 2.76**	**2.31**	**2.10, 2.55**
**Current e-cigarette use**						
No	–	–	–	–	–	–
Yes	**4.99**	**4.59, 5.43**	**4.67**	**4.28, 5.09**	**4.79**	**4.51, 5.09**
**Current cannabis use**						
No	–	–	–	–	–	–
Yes	**4.00**	**3.60, 4.45**	**3.34**	**3.02, 3.70**	**3.65**	**3.39, 3.92**
**Ever-use opiates**						
No	–	–	–	–	–	–
Yes	**4.65**	**2.62, 8.27**	**5.12**	**3.52, 7.46**	**4.60**	**3.37, 6.29**
**Outside of school sport**						
No	–	–	–	–	–	–
Yes	**1.18**	**1.08, 1.29**	**1.38**	**1.26, 1.50**	**1.28**	**1.20, 1.36**
**Competitive school sport**						
No	–	–	–	–	–	–
Yes	**1.47**	**1.32, 1.64**	**1.49**	**1.35, 1.65**	**1.47**	**1.37, 1.58**
**MVPA guidelines**						
No	–	–	–	–	–	–
Yes	**1.10**	**1.01, 1.21**	**1.24**	**1.14, 1.35**	**1.20**	**1.13, 1.27**
**Non-competitive school sport**						
No	–	–	–	–	–	–
Yes	1.05	0.95, 1.17	**1.18**	**1.07, 1.30**	**1.12**	**1.04, 1.20**

OR, odds ratio, CI, confidence interval, Bold, BH adjusted p-value < 0.05. MVPA, moderate to vigorous physical activity. GAD-7, generalized anxiety disorder-7.

There were no significant interaction effects for females or males for school connectedness by sport participation or self-reported MVPA for current binge drinking.

## Discussion

This study examined the association between physical activity, sport participation, and substance use in Canadian adolescents. E-cigarette use (26% of females and 31% of males) was the most prevalent current substance use behavior, followed by binge drinking (16% females and 19% males). Participation in sport outside-of-school had the most participation with 37% of females and 45% of males. Less than half of males and less than a third of females self-reported at least 60 min of MVPA per day. Generally, all types of sport participation were associated with current substance use behavior. Less evidence was found for the association between meeting physical activity guideline recommendations and current substance use. This suggests that the mechanisms underpinning any effect of physical activity participation are likely inextricably linked to the sport context and culture in which physical activity may be accumulated. Differences in the direction of the associations were observed more commonly between substances (i.e., cigarette smoking versus alcohol) rather than between types of physical activities (i.e., non-competitive school sport vs. competitive school sport). However, directional differences were observed between different types of sport participation and their associations with e-cigarette use for females. Finally, modest evidence was found for the potential role of school connectedness in moderating the relationship between sport participation or 60 min of MVPA/day and substance use. High school connectedness appeared protective when interacting with sport participation, particularly for males regarding e-cigarette use.

The results are consistent with those reported by Williams and colleagues (21). All types of sport participation were either not associated or negatively associated with current smoking and cannabis use. Potential explanations for these results may be that non-competitive sport is typically under the supervision of a teacher, and perhaps the social peer connections made during this time reinforce ideas about healthy living, which may be connected to the social type of influence at the proximal level in the Petraitis et al. (13) conceptual framework (e.g., motivation to comply with others). For competitive school sport and outside-of-school sport, the negative associations may be due to the perceived harmfulness of smoking cigarettes or cannabis to athletic performance and accountability from teammates to perform at their highest level (48).

Consistent with our hypothesis, all types of sport participation were generally positively associated with current binge drinking behavior. Potential explanations for these associations may be the normative culture of certain team sports that foster an environment of competitiveness and masculinity (49, 50). Further, it has been found that alcohol has been used as part of hazing rituals for sport teams during adolescence and young adulthood (51). However, it is important to note that non-competitive school sport participation for females was not associated with current binge drinking. It is possible that for females, non-competitive school sport does not have the detrimental team dynamics or environment that is outlined above.

Notably, the most prevalent substance use was e-cigarette use. This reflects increasing public health concerns about this behavior and the need for regulatory frameworks to shift e-cigarette use away from adolescents (52). Our results indicate that non-competitive school sport participation was negatively associated with current e-cigarette use. In contrast, competitive school sport and outside-of-school sport were positively associated with current e-cigarette use for females. Similar results were found in the COMPASS data (year 6: 2017–2018) (21). It is important to consider why non-competitive school sport participation was negatively associated with e-cigarette use, whereas the other modalities of sport participation were positively associated. Competitive school sports support a team dynamic where peer pressure may be a factor similar to binge drinking (51). Finally, the positive associations for sport participation may be due to the perceptions that the dangers of e-cigarette use may not be as severe as traditional cigarette smoking. Hence, the behavior is not seen as detrimental to their athletic performance (53).

To the authors' knowledge, no study differentiates the three distinct types of sport participation that our analysis considers. The findings suggest that the psychosocial mechanisms underpinning the associations with substance use may be similar for outside-of-school and competitive school sports. Both outside-of-school sport and competitive school sport typically require a higher level of skill and motivation to play, and perhaps individuals with higher competitiveness choose to participate (54). The interplay between these factors may explain why both outside- of-school sport and competitive school sport participation are positively associated with the use of some substances, particularly alcohol use. In contrast, non-competitive school sport participation generally had a more favorable set of negative or null associations with substance use. Non-competitive school sport is likely less structured and less competitive in nature, and may involve a broader range of non-traditional activities (e.g., ultimate frisbee) compared to traditional competitive school sports in Canada (e.g., basketball). Thus, the nature of the sport context, and likely the adolescents attracted to these different contexts, may be very different.

Controlling for sport participation, the negative associations with on current substance use hypothesized for a adolescents self-reporting more than 60 min of daily MVPA were not found within this sample. While inconsistent with some of the previous literature (19, 55–58), there is also evidence to suggest that physical activity may vary in its association with substance use, particularly with alcohol use (59). The correlates of physical activity co-vary with correlates of substance use in adolescents, leading to heterogeneous results. For example, extraversion and openness as a personality type, and the ease of influence by peers have been related to increasing both physical activity and substance use (13, 60–64). Differences in findings may be explained by how physical activity is operationalized and measured. As with sport participation, future research that better characterizes the ‘dose’ of sport participation or physical activity (e.g., frequency, intensity, time, type) is needed.

Our speculation was that the interplay of sport participation and school connectedness would enhance negative associations and mitigate positive associations. However, we found modest evidence for this through our analysis. When considering current e-cigarette use, our analysis identified two significant interaction effects for males (outside-of-school sport and MVPA level by school connectedness). This result provides evidence for the potential of the moderation between physical activities and school connectedness to attenuate the risk factors for current e-cigarette use. This is consistent with our hypothesis as the model by Petraitis et al. (13) provides evidence that school connectedness may protect against adolescent substance use at a distal level. School connectedness may be an important factor to consider when creating interventions to prevent e-cigarette use among adolescents in the future (65, 66).

Despite this finding, more robust evidence for this moderation was expected. It is possible that other unmeasured factors may be stronger moderators of the association between physical activity and substance use. For example, peer and parental relationships have been shown to be important within the literature (67, 68) and in the conceptual framework of Petraitis et al. (13). However, irrespective of the interaction effect seen, Figures 1–5 illustrate, individuals who are participating in sport (or not) with high school connectedness are at a lower risk of reporting current substance use. Schools have a role in fostering school connectedness through sport, and future work should attempt to assess if school connectedness mediates a prospective relationship between sport participation and substance use.

### Strengths and limitations

The strengths and limitations of this study should be acknowledged. First, this sample consists of data from over 50,000 students in Canada. However, the COMPASS study utilizes a convenience sampling approach and is not representative of all teens in Canada. The cross-sectional design of our study does not allow for causation to be determined. The study was based on self-reported measures, which may lead to bias. Additionally, the COMPASS study assessed the gender variable by asking “Are you male or female?” and provides male, female, I do not identify with those genders, prefer not to answer as the available options. It is typical that male and female are used for the collection of sex rather than gender (43). Where possible, the measures are validated and are the research standard for Canadian epidemiological studies (36, 39). Our study covaries several key variables associated with substance use and physical activity/sport participation in adolescents (13, 69). This study also employed the BH adjustment controlling for false discovery rate, which decreases the likelihood of a type 1 error (44). The measures used for sports participation lack validity and reliability and are somewhat crude in design by not, for example, differentiating the types of sport played duration or frequency of practice and performances, or level of competition. As with the broader literature, most epidemiological research in this area draws on surveys where interest in sport participation is not central (57, 70–72).

## Conclusion

Our study provides evidence for further consideration of extracurricular activities, such as non-competitive school sport, for lessening the odds of current substance use. In the context of our sample, approximately one-third of students participate in non-competitive school sport, and it may be advantageous to increase the offering of this type of programming to adolescents. Caution is required in claiming that sport participation or physical activity, in general, is negatively associated with substance use. Rather, it would appear that sport programmers should be considering more deliberate efforts to educate about the risks of substance use and build a normative culture that inhibits substance use. Students reporting high levels of school connectedness do appear to be at lower risk in the use of some substances (e-cigarette use) that are associated with sport participation. School connectedness should remain an important target for intervention by school staff and administrators for its broad benefits (28, 73, 74).

## Data availability statement

The data analyzed in this study is subject to the following licenses/restrictions: https://uwaterloo.ca/compass-system/information-researchers “Data from the COMPASS study is stored at the University of Waterloo on a secure server. The Principal Investigator of COMPASS (Dr. SL) maintains ownership of all COMPASS data. Access to specified COMPASS data may be granted to all COMPASS project collaborators and/or their research teams or students as well as external researchers/teams or students. Special preference will be granted to graduate student and internal collaborator requests. In order for any researcher/team/student to gain access to the COMPASS data, the successful completion and approval of the COMPASS Data Usage Application is required. The application will be reviewed and approved/declined by the Principal Investigator of COMPASS.” Requests to access these datasets should be directed to Katie Burns (katie.burns@uwaterloo.ca).

## Ethics statement

The studies involving human participants were reviewed and approved by the University of Waterloo Office of Research Ethics, the University of British Columbia's Research Ethics Board, and Appropriate School Board Committees. Written informed consent from the participants' legal guardian/next of kin was not required to participate in this study in accordance with the national legislation and the institutional requirements.

## Author contributions

MF, RB, EP, SL, and GF contributed to conception and design of the study. SL organized the database and lead the study data collection. MF and MD performed the statistical analysis. MF wrote the first draft of the manuscript. MF, MD, RB, EP, SL, and GF wrote sections of the manuscript. All authors contributed to manuscript revision, read, and approved the submitted version.

## Funding

The COMPASS study has been supported by a bridge grant from the CIHR Institute of Nutrition, Metabolism and Diabetes (INMD) through the Obesity—Interventions to Prevent or Treat priority funding awards (OOP-110788; awarded to SL), an operating grant from the CIHR Institute of Population and Public Health (IPPH) (MOP-114875; awarded to SL), a CIHR project grant (PJT-148562; awarded to SL), a CIHR bridge grant (PJT-149092; awarded to KP/SL), a CIHR project grant (PJT-159693; awarded to KP), and by a research funding arrangement with Health Canada (#1617-HQ-000012; contract awarded to SL), a CIHR-Canadian Center on Substance Abuse (CCSA) team grant (OF7 B1-PCPEGT 410-10-9633; awarded to SL), and a SickKids Foundation New Investigator Grant, in partnership with CIHR Institute of Human Development, Child and Youth Health (IHDCYH) (Grant No. NI21-1193; awarded to KAP) funds a mixed methods study examining the impact of the COVID-19 pandemic on youth mental health, leveraging COMPASS study data. The COMPASS-Quebec project additionally benefits from funding from the Ministère de la Santé et des Services sociaux of the province of Québec, and the Direction régionale de santé publique du CIUSSS de la Capitale-Nationale. MJF was supported by a CIHR Studentship.

## Conflict of interest

The authors declare that the research was conducted in the absence of any commercial or financial relationships that could be construed as a potential conflict of interest.

## Publisher's note

All claims expressed in this article are solely those of the authors and do not necessarily represent those of their affiliated organizations, or those of the publisher, the editors and the reviewers. Any product that may be evaluated in this article, or claim that may be made by its manufacturer, is not guaranteed or endorsed by the publisher.
