# Supplementary material for: Physical activity and substance use among Canadian adolescents: Examining the moderating role of school connectedness
Source: Front Public Health. 2022 Nov 10;10:889987. doi: 10.3389/fpubh.2022.889987 (PMC9686278; doi:10.3389/fpubh.2022.889987)
Supplement: Supplementary file 1 [file Data_Sheet_1.PDF]

Supplementary file

## Table of Contents


**Table S1: Model fit statistics for current cigarette use by predictor ........ 2**

**Table S2: Model fit statistics for current e-cigarette use by predictor ........ 3**

**Table S3: Model fit statistics for current cannabis use by predictor ........ 4**

**Table S4: Model fit statistics for current binge drinking by predictor ........ 5**

**Table S5: Male stratified school connectedness and MVPA moderation model for current cigarette use ........ 6**

**Table S6: Male stratified school connectedness and outside of school sport participation moderation for e-cigarette use ........ 8**

**Table S7: Male stratified school connectedness and MVPA moderation for e-cigarette use ........ 10**

**Table S8: Female stratified school connectedness and MVPA moderation for cannabis use ........ 12**

**Table S9: Male stratified school connectedness and outside of school sport moderation for cannabis use ........ 14**

Supplementary file

## Table S1: Model fit statistics for current cigarette use by predictor

| | Non- competitive sport | | Competitive sport | | Outside of School sport | | MVPA guidelines | |
|---|---|---|---|---|---|---|---|---|
| | Male | Female | Male | Female | Male | Female | Male | Female |
| ICC= | 0.122 | 0.128 | 0.122 | 0.128 | 0.122 | 0.128 | 0.122 | 0.128 |
| Step # | AIC | AIC | AIC | AIC | AIC | AIC | AIC | AIC |
| Step 1 | 19996.3 | 17214.4 | 19996.3 | 17214.4 | 19996.3 | 17214.4 | 19996.3 | 17214.4 |
| Step 2 | 18408.5 | 16030.6 | 18920.6 | 16467.0 | 18973.7 | 16448.7 | 19368 | 16751.7 |
| Step 3 | 14772.3 | 12882.7 | 15184.6 | 13228.8 | 15238.5 | 13232.9 | 15555.9 | 15555.9 |
| Step 4 | 8565.5 | 8025.0 | 8744.2 | 8245.8 | 8820.2 | 8234.5 | 8963.1 | 8356.7 |
| Step 5 | 8140.8 | 7642.5 | 8140.8 | 7642.5 | 8140.8 | 7642.5 | 8140.8 | 7642.5 |

*Notes*. ICC=intraclass coefficient, AIC= Akaike information criterion. MVPA=moderate-to-vigorous physical activity. Step 1= base model, step 2=predictor of interest, step 3= demographic covariates, step 4 = social and mental health variables, step 5 =controlling for other sport participation and MVPA guidelines.

Table S2: Model fit statistics for current e-cigarette use by predictor

| | Non- competitive sport | | Competitive sport | | Outside of School sport | | MVPA guidelines | |
|---|---|---|---|---|---|---|---|---|
| | Male | Female | Male | Female | Male | Female | Male | Female |
| ICC= | 0.068 | 0.063 | 0.068 | 0.063 | 0.068 | 0.063 | 0.068 | 0.063 |
| Step # | AIC | AIC | AIC | AIC | AIC | AIC | AIC | AIC |
| Step 1 | 43817.3 | 40378 | 43817.3 | 40378 | 43817.3 | 40378 | 43817.3 | 40378 |
| Step2 | 41722.5 | 38640.4 | 42449.6 | 39383.3 | 42699.2 | 39565.1 | 39655.7 | 39655.7 |
| Step 3 | 32731.2 | 30952.6 | 33285.3 | 31540.7 | 33417.7 | 31672.2 | 33613.6 | 31826.6 |
| Step 4 | 23062.7 | 23414.7 | 23481.0 | 23808.3 | 23572.9 | 23909.6 | 25437 | 24027.6 |
| Step 5 | 21657.4 | 22330.5 | 21657.4 | 22330.5 | 21657.4 | 22330.5 | 21657.4 | 22330.5 |

*Notes*. ICC=intraclass coefficient, AIC= Akaike information criterion. MVPA=moderate-to-vigorous physical activity. Step 1= base model, step 2=predictor of interest, step 3= demographic covariates, step 4 = social and mental health variables, step 5 =controlling for other sport participation and MVPA guidelines.

## Table S3: Model fit statistics for current cannabis use by predictor

| | Non- competitive sport | | Competitive sport | | Outside of School sport | | MVPA guidelines | |
|---|---|---|---|---|---|---|---|---|
| | Male | Female | Male | Female | Male | Female | Male | Female |
| ICC= | 0.103 | 0.122 | 0.103 | 0.122 | 0.103 | 0.122 | 0.103 | 0.122 |
| Step # | AIC | AIC | AIC | AIC | AIC | AIC | AIC | AIC |
| Step 1 | 29723.8 | 24301.0 | 29723.8 | 24301.0 | 29723.8 | 24301.0 | 29723.8 | 24301.0 |
| Step2 | 27948.1 | 22961.7 | 28553.9 | 23503.9 | 28685.9 | 23499.0 | 28884.4 | 23796.1 |
| Step 3 | 23213.9 | 19644.1 | 23696.3 | 20065.9 | 23792.8 | 20069.8 | 23986.1 | 20331.3 |
| Step 4 | 14295.3 | 12899.8 | 14599.1 | 13147.6 | 14649.1 | 13128.5 | 14764.7 | 13280.8 |
| Step 5 | 13372.1 | 11970.9 | 13372.1 | 11970.9 | 13372.1 | 11970.9 | 13372.1 | 11970.9 |

*Notes*. ICC=intraclass coefficient, AIC= Akaike information criterion. MVPA=moderate-to-vigorous physical activity. Step 1= base model, step 2=predictor of interest, step 3= demographic covariates, step 4 = social and mental health variables, step 5 =controlling for other sport participation and MVPA guidelines.

Table S4: Model fit statistics for current binge drinking by predictor

| | Non- competitive sport | | Competitive sport | | Outside of School sport | | MVPA guidelines | |
|---|---|---|---|---|---|---|---|---|
| | Male | Female | Male | Female | Male | Female | Male | Female |
| ICC= | 0.101 | 0.072 | 0.101 | 0.072 | 0.101 | 0.072 | 0.101 | 0.072 |
| Step # | AIC | AIC | AIC | AIC | AIC | AIC | AIC | AIC |
| Step 1 | 34612.2 | 30993.1 | 34612.2 | 30993.1 | 34612.2 | 30993.1 | 34612.2 | 30993.1 |
| Step 2 | 32756.3 | 29523.0 | 33201.4 | 30103.4 | 33506.2 | 30242.7 | 33663.4 | 30422.1 |
| Step 3 | 25546.2 | 23826.7 | 25856.5 | 24257.6 | 26011.4 | 24360.9 | 26319.3 | 24582.5 |
| Step 4 | 17662.2 | 17209.3 | 17966.3 | 17539.6 | 18044.7 | 17604.0 | 17728.2 | 17728.2 |
| Step 5 | 16749.6 | 16532.7 | 16749.6 | 16532.7 | 16749.6 | 16532.7 | 16749.6 | 16532.7 |

*Notes*. ICC=intraclass coefficient, AIC= Akaike information criterion. MVPA=moderate-to-vigorous physical activity. Step 1= base model, step 2=predictor of interest, step 3= demographic covariates, step 4 = social and mental health variables, step 5 =controlling for other sport participation and MVPA guidelines.

Table S5: Male stratified school connectedness and MVPA moderation model for current cigarette use

| Characteristic | OR | 95% CI | p-value | BH p-value |
|---|---|---|---|---|
| **Grade** | | | | |
| 9 | — | — | | |
| 10 | 1.02 | 0.85, 1.22 | 0.8 | 0.84 |
| 11 | 1.23 | 1.03, 1.46 | **0.025** | **0.037** |
| 12 | 1.53 | 1.26, 1.87 | **<0.001** | **<0.001** |
| **Ethnicity** | | | | |
| White | — | — | | |
| Mixed | 1.31 | 1.05, 1.63 | **0.017** | **0.03** |
| Black | 1.62 | 1.20, 2.18 | **0.002** | **0.003** |
| Asian | 1.15 | 0.88, 1.51 | 0.3 | 0.35 |
| Latin | 1.27 | 0.89, 1.81 | 0.2 | 0.23 |
| Other | 1.96 | 1.59, 2.42 | **<0.001** | **<0.001** |
| **Pocket money** | | | | |
| None | — | — | | |
| 1-20$ | 1.07 | 0.85, 1.36 | 0.6 | 0.58 |
| 21-100$ | 1.10 | 0.88, 1.37 | 0.4 | 0.47 |
| >100$ | 1.28 | 1.03, 1.59 | **0.024** | **0.037** |
| "I don't know" | 1.08 | 0.84, 1.40 | 0.5 | 0.577 |
| **GAD-7** | | | | |
| 0 (minimal) | — | — | | |
| 1 (mild) | 1.19 | 1.04, 1.37 | **0.013** | **0.024** |
| 2 (Moderate) | 1.36 | 1.13, 1.64 | **0.001** | **0.003** |
| 3 (severe) | 1.63 | 1.31, 2.01 | **<0.001** | **<0.001** |
| **School connectedness** | 0.93 | 0.91, 0.95 | **<0.001** | **<0.001** |
| **Current e-cigarette use** | | | | |
| No | — | — | | |
| Yes | 7.61 | 6.43, 9.00 | **<0.001** | **<0.001** |
| **Current cannabis use** | | | | |
| No | — | — | | |
| Yes | 3.93 | 3.45, 4.48 | **<0.001** | **<0.001** |
| **Current binge drinking** | | | | |
| No | — | — | | |

| Characteristic | OR | 95% CI | p-value | BH p-value |
|---|---|---|---|---|
| Yes | 2.63 | 2.30, 3.00 | **<0.001** | **<0.001** |
| **Ever-use opiates** | | | | |
| No | — | — | | |
| Yes | 4.77 | 3.42, 6.64 | **<0.001** | **<0.001** |
| **Competitive school sport** | | | | |
| No | — | — | | |
| Yes | 0.67 | 0.57, 0.78 | **<0.001** | **<0.001** |
| **Non-competitive school sport** | | | | |
| No | — | — | | |
| Yes | 0.88 | 0.76, 1.03 | 0.11 | 0.146 |
| **Outside of school sport** | | | | |
| No | — | — | | |
| Yes | 0.72 | 0.63, 0.82 | **<0.001** | **<0.001** |
| **MVPA guidelines** | | | | |
| No | — | — | | |
| Yes | 0.61 | 0.34, 1.09 | 0.10 | 0.132 |
| **School connectedness x MVPA guidelines** | | | | |
| School connectedness x no | | | | |
| School connectedness x yes | 1.04 | 1.00, 1.07 | **0.035** | **0.049** |

*Notes*. OR = Odds Ratio, CI = Confidence Interval, **Bold=** p-value <0.05.
MVPA=Moderate to vigorous physical activity. GAD-7=Generalized anxiety disorder-7.

Table S6: Male stratified school connectedness and outside of school sport participation moderation for e-cigarette use

| Characteristic | OR | 95% CI | p-value | BH p-value |
|---|---|---|---|---|
| **Grade** | | | | |
| 9 | — | — | | |
| 10 | 1.07 | 0.98, 1.17 | 0.12 | 0.146 |
| 11 | 1.06 | 0.96, 1.16 | 0.2 | 0.265 |
| 12 | 0.88 | 0.78, 0.99 | **0.029** | **0.037** |
| **Ethnicity** | | | | |
| White | — | — | | |
| Mixed | 0.82 | 0.71, 0.93 | **0.003** | **0.004** |
| Black | 0.59 | 0.49, 0.72 | **<0.001** | **<0.001** |
| Asian | 0.57 | 0.49, 0.66 | **<0.001** | **<0.001** |
| Latin | 0.93 | 0.75, 1.14 | 0.5 | 0.515 |
| Other | 0.79 | 0.68, 0.91 | **0.001** | **0.002** |
| **Pocket money** | | | | |
| None | — | — | | |
| 1-20$ | 1.45 | 1.28, 1.63 | **<0.001** | **<0.001** |
| 21-100$ | 1.93 | 1.71, 2.16 | **<0.001** | **<0.001** |
| >100$ | 2.19 | 1.95, 2.47 | **<0.001** | **<0.001** |
| "I don't know" | 1.32 | 1.16, 1.51 | **<0.001** | **<0.001** |
| **GAD-7** | | | | |
| 0 (minimal) | — | — | | |
| 1 (mild) | 1.35 | 1.25, 1.47 | **<0.001** | **<0.001** |
| 2 (Moderate) | 1.24 | 1.10, 1.40 | **<0.001** | **<0.001** |
| 3 (severe) | 1.22 | 1.05, 1.42 | **0.010** | **0.014** |
| **School connectedness** | 1.00 | 0.98, 1.01 | 0.7 | 0.748 |
| **Current cigarette use** | | | | |
| No | — | — | | |
| Yes | 6.55 | 5.51, 7.79 | **<0.001** | **<0.001** |
| **Current cannabis use** | | | | |
| No | — | — | | |
| Yes | 7.24 | 6.52, 8.04 | **<0.001** | **<0.001** |
| **Current binge drinking** | | | | |
| No | — | — | | |

| Characteristic | OR | 95% CI | p-value | BH p-value |
|---|---|---|---|---|
| Yes | 4.49 | 4.12, 4.90 | **<0.001** | **<0.001** |
| **Ever-use opiates** | | | | |
| No | — | — | | |
| Yes | 1.02 | 0.65, 1.61 | >0.9 | 0.928 |
| **Competitive school sport** | | | | |
| No | — | — | | |
| Yes | 1.41 | 1.29, 1.54 | **<0.001** | **<0.001** |
| **Non-competitive school sport** | | | | |
| No | — | — | | |
| Yes | 0.92 | 0.85, 1.00 | 0.052 | 0.064 |
| **MVPA guidelines** | | | | |
| No | — | — | | |
| Yes | 1.16 | 1.08, 1.24 | **<0.001** | **<0.001** |
| **Outside of school sport** | | | | |
| No | — | — | | |
| Yes | 2.74 | 1.84, 4.08 | **<0.001** | **<0.001** |
| **School connectedness x outside of school sport** | | | | |
| School connectedness x no | | | | |
| School connectedness x yes | 0.96 | 0.94, 0.98 | **<0.001** | **<0.001** |

*Notes*. OR = Odds Ratio, CI = Confidence Interval, **Bold=** p-value <0.05. MVPA=Moderate to vigorous physical activity. GAD-7=Generalized anxiety disorder-7.

Table S7: Male stratified school connectedness and MVPA moderation for e-cigarette use

| Characteristic | OR | 95% CI | p-value | BH p-value |
|---|---|---|---|---|
| **Grade** | | | | |
| 9 | — | — | | |
| 10 | 1.07 | 0.98, 1.17 | 0.13 | 0.15 |
| 11 | 1.06 | 0.96, 1.16 | 0.2 | 0.27 |
| 12 | 0.87 | 0.78, 0.98 | **0.025** | **0.032** |
| **Ethnicity** | | | | |
| White | — | — | | |
| Mixed | 0.82 | 0.71, 0.93 | **0.003** | **0.004** |
| Black | 0.60 | 0.49, 0.72 | **<0.001** | **<0.001** |
| Asian | 0.58 | 0.50, 0.66 | **<0.001** | **<0.001** |
| Latin | 0.93 | 0.75, 1.14 | 0.5 | 0.504 |
| Other | 0.79 | 0.68, 0.92 | **0.002** | **0.003** |
| **Pocket money** | | | | |
| None | — | — | | |
| 1-20$ | 1.45 | 1.29, 1.64 | **<0.001** | **<0.001** |
| 21-100$ | 1.93 | 1.72, 2.17 | **<0.001** | **<0.001** |
| >100$ | 2.20 | 1.96, 2.48 | **<0.001** | **<0.001** |
| “I don’t know” | 1.33 | 1.17, 1.52 | **<0.001** | **<0.001** |
| **GAD-7** | | | | |
| 0 (minimal) | — | — | | |
| 1 (mild) | 1.36 | 1.25, 1.47 | **<0.001** | **<0.001** |
| 2 (Moderate) | 1.24 | 1.10, 1.40 | **<0.001** | **<0.001** |
| 3 (severe) | 1.21 | 1.04, 1.41 | **0.014** | **0.02** |
| **School connectedness** | 0.99 | 0.98, 1.01 | 0.3 | 0.29 |
| **Current cigarette use** | | | | |
| No | — | — | | |
| Yes | 6.49 | 5.46, 7.71 | **<0.001** | **<0.001** |
| **Current cannabis use** | | | | |
| No | — | — | | |
| Yes | 7.21 | 6.49, 8.00 | **<0.001** | **<0.001** |
| **Current binge drinking** | | | | |
| No | — | — | | |
| Yes | 4.51 | 4.13, 4.91 | **<0.001** | **<0.001** |

| Characteristic | OR | 95% CI | p-value | BH p-value |
|---|---|---|---|---|
| **Ever-use opiates** | | | | |
| No | — | — | | |
| Yes | 1.01 | 0.64, 1.60 | >0.9 | >0.9 |
| **Competitive school sport** | | | | |
| No | — | — | | |
| Yes | 1.41 | 1.29, 1.54 | **<0.001** | **<0.001** |
| **Non-competitive school sport** | | | | |
| No | — | — | | |
| Yes | 0.92 | 0.85, 1.00 | 0.055 | 0.067 |
| **Outside of school sport** | | | | |
| No | — | — | | |
| Yes | 1.29 | 1.19, 1.39 | **<0.001** | **<0.001** |
| **MVPA guidelines** | | | | |
| No | — | — | | |
| Yes | 1.82 | 1.24, 2.69 | **0.002** | **0.004** |
| **School connectedness x MVPA guidelines** | | | | |
| School connectedness x no | | | | |
| School connectedness x yes | 0.98 | 0.96, 1.00 | **0.019** | **0.0262** |

*Notes*. OR = Odds Ratio, CI = Confidence Interval, **Bold=** p-value <0.05. MVPA=Moderate to vigorous physical activity. GAD-7=Generalized anxiety disorder-7.

Table S8: Female stratified school connectedness and MVPA moderation for cannabis use

| Characteristic | OR | 95% CI | p-value | BH p-value |
|---|---|---|---|---|
| **Grade** | | | | |
| 9 | — | — | | |
| 10 | 1.29 | 1.12, 1.50 | **<0.001** | **<0.001** |
| 11 | 1.66 | 1.43, 1.92 | **<0.001** | **<0.001** |
| 12 | 1.91 | 1.62, 2.25 | **<0.001** | **<0.001** |
| **Ethnicity** | | | | |
| White | — | — | | |
| Mixed | 1.15 | 0.97, 1.36 | 0.11 | 0.12 |
| Black | 1.58 | 1.21, 2.06 | **<0.001** | **<0.001** |
| Asian | 0.50 | 0.40, 0.64 | **<0.001** | **<0.001** |
| Latin | 0.87 | 0.65, 1.18 | 0.4 | 0.41 |
| Other | 1.52 | 1.26, 1.84 | **<0.001** | **<0.001** |
| **Pocket money** | | | | |
| None | — | — | | |
| 1-20$ | 1.44 | 1.18, 1.74 | **<0.001** | **<0.001** |
| 21-100$ | 1.49 | 1.24, 1.79 | **<0.001** | **<0.001** |
| >100$ | 1.60 | 1.33, 1.94 | **<0.001** | **<0.001** |
| "I don't know" | 1.10 | 0.88, 1.36 | 0.4 | 0.41 |
| **GAD-7** | | | | |
| 0 (minimal) | — | — | | |
| 1 (mild) | 1.22 | 1.06, 1.39 | **0.004** | **0.005** |
| 2 (Moderate) | 1.43 | 1.24, 1.66 | **<0.001** | **<0.001** |
| 3 (severe) | 1.63 | 1.40, 1.89 | **<0.001** | **<0.001** |
| **School connectedness** | 0.88 | 0.86, 0.90 | **<0.001** | **<0.001** |
| **Current cigarette use** | | | | |
| No | — | — | | |
| Yes | 4.72 | 4.12, 5.42 | **<0.001** | **<0.001** |
| **Current e-cigarette use** | | | | |
| No | — | — | | |
| Yes | 4.96 | 4.46, 5.52 | **<0.001** | **<0.001** |
| **Current binge drinking** | | | | |
| No | — | — | | |
| Yes | 4.16 | 3.74, 4.63 | **<0.001** | **<0.001** |

| Characteristic | OR | 95% CI | p-value | BH p-value |
|---|---|---|---|---|
| **Ever-use opiates** | | | | |
| No | — | — | | |
| Yes | 11.6 | 5.65, 24.0 | **<0.001** | **<0.001** |
| **Competitive school sport** | | | | |
| No | — | — | | |
| Yes | 1.02 | 0.89, 1.16 | 0.8 | 0.81 |
| **Non-competitive school sport** | | | | |
| No | — | — | | |
| Yes | 0.80 | 0.70, 0.91 | **<0.001** | **0.001** |
| **Outside of school sport** | | | | |
| No | — | — | | |
| Yes | 0.78 | 0.70, 0.87 | **<0.001** | **<0.001** |
| **MVPA guidelines** | | | | |
| No | — | — | | |
| Yes | 0.54 | 0.32, 0.92 | **0.023** | **0.026** |
| **School connectedness x MVPA guidelines** | | | | |
| School connectedness x no | | | | |
| School connectedness x yes | 1.04 | 1.01, 1.07 | **0.008** | **0.01** |

*Notes*. OR = Odds Ratio, CI = Confidence Interval, **Bold=** p-value <0.05. MVPA=Moderate to vigorous physical activity. GAD-7=Generalized anxiety disorder-7.

Table S9: Male stratified school connectedness and outside of school sport moderation for cannabis use

| Characteristic | OR | 95% CI | p-value | BH p-value |
|---|---|---|---|---|
| **Grade** | | | | |
| 9 | — | — | | |
| 10 | 1.40 | 1.22, 1.60 | **<0.001** | **<0.001** |
| 11 | 1.68 | 1.47, 1.92 | **<0.001** | **<0.001** |
| 12 | 2.25 | 1.94, 2.62 | **<0.001** | **<0.001** |
| **Ethnicity** | | | | |
| White | — | — | | |
| Mixed | 1.29 | 1.09, 1.52 | **0.003** | .003 |
| Black | 1.84 | 1.47, 2.29 | **<0.001** | **<0.001** |
| Asian | 0.57 | 0.47, 0.71 | **<0.001** | **<0.001** |
| Latin | 0.98 | 0.74, 1.28 | 0.9 | 0.85 |
| Other | 1.65 | 1.39, 1.96 | **<0.001** | **<0.001** |
| **Pocket money** | | | | |
| None | — | — | | |
| 1-20$ | 1.19 | 1.00, 1.41 | 0.052 | 0.061 |
| 21-100$ | 1.51 | 1.28, 1.77 | **<0.001** | **<0.001** |
| >100$ | 1.54 | 1.31, 1.80 | **<0.001** | **<0.001** |
| "I don't know" | 1.09 | 0.90, 1.32 | 0.4 | 0.39 |
| **GAD-7** | | | | |
| 0 (minimal) | — | — | | |
| 1 (mild) | 1.14 | 1.02, 1.26 | **0.016** | **0.020** |
| 2 (Moderate) | 1.26 | 1.09, 1.47 | **0.002** | **0.002** |
| 3 (severe) | 1.32 | 1.11, 1.58 | **0.002** | **0.003** |
| **School connectedness** | 0.91 | 0.89, 0.93 | **<0.001** | **<0.001** |
| **Current cigarette use** | | | | |
| No | — | — | | |
| Yes | 7.83 | 7.05, 8.70 | **<0.001** | **<0.001** |
| **Current e-cigarette use** | | | | |
| No | — | — | | |
| Yes | 3.75 | 3.29, 4.27 | **<0.001** | **<0.001** |
| **Current binge drinking** | | | | |
| No | — | — | | |
| Yes | 3.32 | 3.00, 3.67 | **<0.001** | **<0.001** |

| Characteristic | OR | 95% CI | p-value | BH p-value |
|---|---|---|---|---|
| **Ever-use opiates** | | | | |
| No | — | — | | |
| Yes | 6.94 | 4.42, 10.9 | **<0.001** | **<0.001** |
| **Competitive school sport** | | | | |
| No | — | — | | |
| Yes | 1.04 | 0.93, 1.17 | 0.5 | 0.49 |
| **Non-competitive school sport** | | | | |
| No | — | — | | |
| Yes | 0.83 | 0.74, 0.93 | **0.002** | **0.003** |
| **MVPA guidelines** | | | | |
| No | — | — | | |
| Yes | 1.09 | 0.99, 1.19 | 0.076 | 0.086 |
| **Outside of school sport** | | | | |
| No | — | — | | |
| Yes | 0.46 | 0.28, 0.75 | **0.002** | **0.003** |
| **School connectedness x outside of school sport** | | | | |
| School connectedness x no | | | | |
| School connectedness x yes | 1.04 | 1.01, 1.07 | **0.005** | **0.007** |

*Notes*. OR = Odds Ratio, CI = Confidence Interval, **Bold=** p-value <0.05. MVPA=Moderate to vigorous physical activity. GAD-7=Generalized anxiety disorder-7.
